# Electron-Deficient Multicenter Bonding in Phase Change Materials: A Chance for Reconciliation

**DOI:** 10.3390/ma17122840

**Published:** 2024-06-11

**Authors:** Francisco Javier Manjón, Hussien H. Osman, Matteo Savastano, Ángel Vegas

**Affiliations:** 1 Instituto de Diseño para la Fabricación y Producción Automatizada, MALTA Consolider Team, Universitat Politècnica de València, 46022 Valencia, Spain; hussien.helmy@uv.es; 2 Instituto de Ciencia de los Materiales de la Universitat de València, MALTA Consolider Team, Universitat de València, 46100 Valencia, Spain; 3 Chemistry Department, Faculty of Science, Helwan University, Cairo 11795, Egypt; 4 Department of Human Sciences for the Promotion of Quality of Life, University San Raffaele Roma, via di Val Cannuta 247, 00166 Rome, Italy; matteo.savastano@uniroma5.it; 5 Universidad de Burgos, Hospital del Rey, 09001 Burgos, Spain; avegasmolina@ubu.es

**Keywords:** phase change materials, hypervalent bonding, metavalent bonding, electron-deficient multicenter bonding

## Abstract

In the last few years, a controversy has been raised regarding the nature of the chemical bonding present in phase change materials (PCMs), many of which are minerals such as galena (PbS), clausthalite (PbSe), and altaite (PbTe). Two opposite bonding models have claimed to be able to explain the extraordinary properties of PCMs in the last decade: the hypervalent (electron-rich multicenter) bonding model and the metavalent (electron-deficient) bonding model. In this context, a third bonding model, the electron-deficient multicenter bonding model, has been recently added. In this work, we comment on the pros and cons of the hypervalent and metavalent bonding models and briefly review the three approaches. We suggest that both hypervalent and metavalent bonding models can be reconciled with the third way, which considers that PCMs are governed by electron-deficient multicenter bonds. To help supporters of the metavalent and hypervalent bonding model to change their minds, we have commented on the chemical bonding in GeSe and SnSe under pressure and in several polyiodides with different sizes and geometries.

## 1. Introduction

Chalcogenides of the *A*^IV^*B*^VI^ and *A*_2_^V^*B*_3_^VI^ families, such as minerals galena (PbS), clausthalite (PbSe), altaite (PbTe), tetradymite (Bi_2_Te_2_S), guanajuatite (Bi_2_Se_3_), tellurantimony (Sb_2_Te_3_), and tellurobismuthite (Bi_2_Te_3_), have attracted the attention of the scientific community for decades since some of the members of these families, recently named incipient metals [1], feature extraordinary properties. The property portfolio of incipient metals includes soft and anharmonic bonds, relatively small band gaps, moderately high electrical conductivity (similar to that of bad metals), high Born effective charges, high dielectric constants, high thermoelectric figures of merit, and high optical absorption [1,2,3,4]. These extraordinary properties make them ideal as phase change materials (PCMs) in data storage applications, as highly efficient thermoelectric and photovoltaic materials for green technologies, and as topological materials for quantum computation.

A big effort has been made to understand the nature of chemical bonding in PCMs for the purpose of designing materials with enhanced properties. For many years, the chemical bonding in PCMs and related materials, such as pnictogens and chalcogens (note that pnictogens are isoelectronic to *A*^IV^*B*^VI^ compounds, some of them being PCMs [5,6,7,8]), was considered to be a rare case of covalent bonding in which a resonant bonding of p-type orbitals occurred [5,6,7,8]. In the last decade, the consensus on the chemical bonding in PCMs has been broken, as shown by the recently published papers [4,9,10,11,12,13,14].

Since 2018, Wuttig and coworkers have considered that PCMs feature a new and unconventional type of bonding, dubbed metavalent bonding. This bonding is different from the covalent resonant bonding present in graphite and benzene and is responsible for the extraordinary properties of PCMs and lead halide perovskites [1,2,3,4,15,16,17,18,19]. By assuming that electrons are fully localized in covalent bonding and fully delocalized in metallic bonding, metavalent bonding was described as a linear or quasi-linear type of two-center one-electron (2c-1e) bonding in which resonant p-type orbitals result in a combination of electrons that are partly localized and partly delocalized, i.e., as an intermediate bonding type between covalent and metallic bonding, hence its name. Therefore, Wuttig and coworkers consider that PCMs are incipient metals with unique properties different from those of covalent and metallic materials.

**Figure 1:**
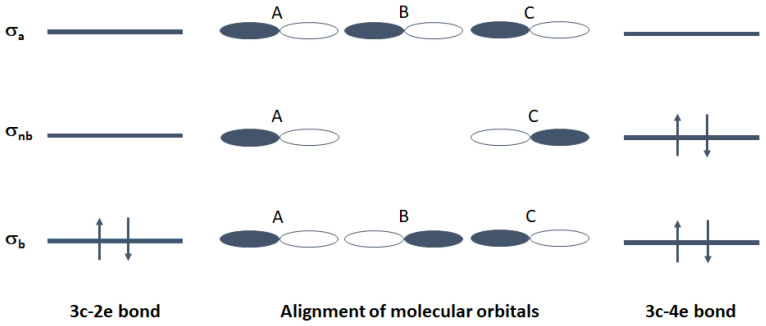
Molecular-orbital energy-level scheme for a 3c-2e EDMB (**left**) and a 3c-4e ERMB (**right**) in an A-B-C trimer linked by p-type electrons (**center**). The bonding (σ_b_), nonbonding (σ_nb_), and antibonding (σ_a_) levels of the three-center bonds are plotted in order of increasing energy. Up and down arrows show the occupation (i) of the lowest-energy molecular orbital with electrons of different spins in the 3c-2e bond (**left**) and (ii) of the two lowest-energy molecular orbitals in the 3c-4e bond (**right**).

On the contrary, other researchers consider that there is no need to claim a new type of bonding since PCMs feature a hypervalent bond [20,21,22,23,24,25,26,27,28], i.e., the old known electron-rich multicenter bond (ERMB), whose most known example is the three-center four-electron (3c-4e) bond. Interestingly, multicenter bonds were originally proposed in the early days of quantum mechanics [29,30,31,32] and were later reformulated in the 1940s and 1950s. Two types of multicenter bonds are known: electron-rich and electron-deficient multicenter bonds. On one hand, the ERMB was modeled as a 3c-4e bond in an A-B-C trimer (see Figure 1 right) by Rundle and Pimentel [33,34]. The ERMB bond is present in many so-called hypervalent molecules of electron-rich elements (groups 15 to 18), such as XeF_2_, XeF_4_, XeF_6_, I_3_^−^, SF_4_, and SF_6_, and it was also proposed to be present in solids with electron-rich elements, e.g., among Sb atoms in simple cubic Sb (sc-Sb) and in the Zintl phases Li_2_Sb and BaZnSb_2_ [35]. On the other hand, the electron-deficient multicenter bond (EDMB) was modeled as a three-center two-electron (3c-2e) bond in an A-B-C trimer (see Figure 1 left) by Rundle and Longuet-Higgings [36,37,38,39]. This bond is typically present in molecules and solids of electron-deficient elements (hydrogen and elements of groups 1, 2, and 13), such as in boranes (B_x_H_y_) and hydrogenonium ion H_3_^+^, and is considered to be a well-established bond type [40], for whose understanding Lipscomb was awarded the Nobel Prize in Chemistry in 1976 [41].

Concerning multicenter bonds, it is commonly believed that EDMBs could only be formed by electron-deficient elements and ERMBs could only be formed by electron-rich elements [42]; however, this is not necessarily true. For instance, hydrogen (an electron-deficient element) can form a 3c-4e ERMB (cataloged as a hydrogen bond by IUPAC) with F atoms in the [FHF]^−^ anion [34,43,44,45]. On the other hand, 3c-2e EDMBs have been found in carbocations (see last chapter of Ref. [40]). Moreover, Vegas and coworkers have suggested that the linear 3c-2e EDMBs should be present in the linear Si-C-Si bond of the carbocation [Si_2_(CH_3_)_7_]^+^, in the linear Si-O-Si bonds of hexamethyldisiloxane, (H_3_C)_3_-Si-O-Si-(CH_3_)_3_, as well as in the linear Si-O-Si bonds within the [O_3_Si-O-SiO_3_]^6−^ polyanion in solid Sc_2_Si_2_O_7_ silicate [46,47].

It is also well known that both 3c-2e EDMBs and 3c-4e ERMBs are “half” or “partial” bonds since both are longer bonds than the single covalent (two-center, two-electron, 2c-2e) bond. For this reason, it has also been believed that both 3c-2e and 3c-4e bonds have a single electron shared between two atoms [42]. In other words, both multicenter bonds have traditionally been considered two different cases of two-center one-electron (2c-1e) bonds. The reason for such a belief is based on the simple molecular orbitals proposed to describe both multicenter bonds in Figure 1. This figure shows that in both 3c-2e and 3c-4e bonds, there are two electrons in the low-energy three-center bonding orbital, thus leading to one shared electron between each of the two atoms. In addition, there are two additional electrons in 3c-4e bonds located at the three-center nonbonding orbital (note that this orbital could have a slight antibonding character, as suggested by Hoffmann and coworkers [48]). These two extra electrons in 3c-4e bonds are considered to be exclusively located in the external atoms of the three-atom molecule (as suggested by the p-type orbitals depicted in the center of Figure 1), so they have not been considered to be even partially shared between the two nearest atoms [42]. In this context, we will show in the next section that the 2c-1e picture of both 3c-2e and 3c-4e bonds seems not to be true for 3c-4e bonds according to recent calculations of the number of electrons shared between centers in molecules with both 3c-2e and 3c-4e bonds.

## 2. Hypervalent vs. Metavalent Bonding Models in PCMs

**Figure 2:**
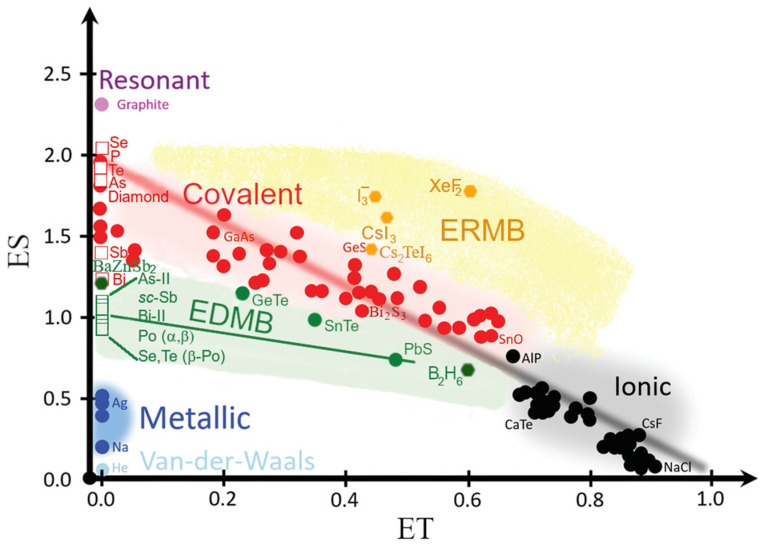
Revisited 2D map of the number of electrons shared (ES) vs. the normalized number of electrons transferred (ET) between two centers, which allows a classification of chemical bonds. The regions of ERMBs (in orange) and EDMBs (in green) are compared to the regions with covalent (in red), ionic (in black), and metallic bonding (in blue). Reproduced with permission from Ref. [2]. Copyright 2021, Wiley-VCH.

The two models for bonding in PCMs (hypervalent and metavalent) are currently under discussion. In a recent study, supporters of the metavalent bonding model calculated the number of electrons shared (ES) and the normalized number of electrons transferred (ET) between two centers (according to Bader’s Quantum Theory of Atoms in Molecules [49]) for typical hypervalent molecules with 3c-4e ERMBs, such as ClF_3_, XeF_2_, and SF_4_ [4]. These hypervalent molecules feature ES values as large as those of covalent 2c-2e bonds, i.e., with almost two shared electrons instead of one. This result is in contrast with the common belief that ERMBs are 2c-1e bonds, as commented in the previous paragraphs. Interestingly, the large ES value of molecules with ERMBs contrasts with the low ES values found in PCMs, such as cubic GeTe, which are close to unity [11], and the less than one shared electron between B-H in the 3c-2e B-H-B bonds in diborane (B_2_H_6_) [12]. Consequently, it has been suggested that bonds in PCMs, such as cubic GeTe, PbS, PbSe, and PbTe, are electron-deficient and not electron-rich bonds [11,12,13]. Moreover, after revisiting the pros and cons of the hypervalent and metavalent bonding models for PCMs, Manjón and coworkers have even suggested that PCMs, as well as the α-Po and β-Po phases of pnictogens and chalcogens, feature not simple electron-deficient bonds but electron-deficient multicenter bonds (EDMBs) [12,13]. The different positions of ERMBs and EDMBs in the ES vs. ET map can be shown in Figure 2. Notice that both PCMs and B_2_H_6_ are electron-deficient bonds and share a similar position in the ES vs. ET map; however, Wuttig and coworkers consider that the electron-deficient bonds present in molecules, such as B_2_H_6_, have nothing to do with those present in solid PCMs [50].

The supporters of the hypervalent bonding model have recently argued that bonds in PCMs cannot be electron-deficient. They have claimed that the ES and ET values are not adequate to classify the different bonding types in materials [9,51]. These authors have argued that density-based methods are not appropriate to characterize bonds and consider that orbital-based methods are required [23,24]. In particular, they have proposed the use of the projected force constants and crystal orbital bond index (COBI) and their integrated values for two and three centers (ICOBI(2c) and ICOBI(3c). In this regard, the supporters of the metavalent bonding model have recently shown that the map obtained from density-based methods (ES vs. ET) is equivalent to the map obtained from orbital-based methods (2·ICOBI(2c) vs. Löwdin charge) [11]—something that, in our opinion, has also been unintentionally shown by the supporters of the hypervalent bonding model [51]. Therefore, in our opinion, it has been demonstrated that density-based methods are as valid as orbital-based methods to classify bonds if we consider that each method has its own limitations and numerical uncertainties.

In addition to the electron-deficient or electron-rich nature of bonding in PCMs, the metavalent and hypervalent bonding models differ in the multicenter character of the interaction. According to the hypervalent bonding model, PCMs feature a multicenter bond that was evidenced by calculations of projected force constants in PCMs and by the calculations of ICOBI(3c) [22,23,24]. The multicenter character of the bonding in PCMs seems to be also supported by recent calculations that model the behavior of a dimerized linear chain under compression [52]. However, the supporters of the metavalent bonding model have not mentioned in their works if bonding in PCMs has a multicenter character. Despite not mentioning the multicenter character of the interaction, the metavalent (electron-deficient) bonding model does not exclude the multicenter character of the interaction. Notice that the multicenter character of electron-deficient half bonds, such as those of PCMs, was already suggested by Rundle (“half-bonds occur in pairs”) more than 70 years ago [36,37]. In summary, the above-commented results on the multicenter character of bonding suggest that bonds in PCMs could likely be one of the two kinds of multicenter bonds already proposed before 1960.

## 3. A New Perspective: The Chance for Reconciliation

In the last few years, Manjón and coworkers have studied the dependence of several compounds under pressure, including several chalcogenides and periodates. Some of the crystalline structures of these materials have been found to exhibit similar properties as PCMs (e.g., Ge-Te in cubic GeTe) and have been tentatively explained with the help of the metavalent bonding model [53,54,55,56,57,58,59] and more recently with the EDMB model [60]. In particular, periodates (with (IO_3_)^−^ units) tend to show hypercoordinated iodine atoms at high pressure that show I-O bonds with the same kind of bonding as PCMs [60]. Similarly, mineral orpiment (As_2_S_3_) tends to show hypercoordinated As atoms at high pressure that show As-S bonds with the same kind of bonding as PCMs [53]. Curiously, the results obtained for orpiment and periodates under compression mimic those previously reported for SnSe and GeSe under compression [61,62].

The important point here is that the results on SnSe, GeSe, and As_2_S_3_ under compression can be extrapolated to other *A*^IV^*B*^VI^ and *A*_2_^V^*B*_3_^VI^ compounds. In particular, the above compounds (also GeS, GeTe, SnS, As_2_Se_3_, As_2_Te_3_, Sb_2_S_3_, Sb_2_Se_3_, and Bi_2_S_3_), which are not PCMs at room pressure, are expected to develop under compression the same kind of bonding observed in PCMs [12,13,53,61,62]. Consequently, the works commented in the previous paragraph have stressed the importance of high pressure to favor the formation of multicenter bonds; an importance that was already suggested [63,64], but later not appropriately addressed.

Following the same line of research, the supporters of the EDMB model have recently studied the simplest materials that can develop at high pressure the same kind of bonding as PCMs, i.e., elemental pnictogens and chalcogens [12,13]. They have evaluated both the perspectives of the metavalent and hypervalent bonding models in PCMs (considering the pros and cons of both bonding models) to explain the properties of pnictogens and chalcogens under compression. They have concluded that, like PCMs, the octahedrally-coordinated phases of pnictogens and chalcogens, such as the α- and β-Po phases, are characterized by EDMBs [12,13]. In other words, they have shown that EDMBs have some features proposed by the supporters of the metavalent and hypervalent bonding models and some features different from those previously proposed by the above two models. Consequently, Manjón and coworkers have proposed the EDMB model to explain the bonding in PCMs and their corresponding properties.

We will now comment on the points of agreement of the EDMB model with the two previous models. In agreement with the supporters of the metavalent bonding model, Manjón and coworkers have suggested that the ES vs. ET map is equivalent to the *f* vs. *c* map of Mori-Sánchez et al. [65], showing that both maps provide valuable parameters to characterize bonds. Notice that other maps can also be used to characterize bonds, e.g., the hybridization (covalency) vs. ionicity map [66] or the electronegativity variation vs. mass density [64]. However, we have preferred to use the ES vs. ET map, which can be calculated from density-based methods and orbital-based methods [11,51], because the electronegativity of elements can vary with pressure [67].

Using the ES vs. ET map (Figure 2), Manjón and coworkers have shown that the ERMBs and EDMBs found in molecules are located in different map locations, which agrees with recently reported results [11,51]. Typically, the ES value of ERMBs is smaller than that of non-polar (ET = 0) covalent bonds but higher than that of polar (ET ≠ 0) single covalent bonds of comparable polarity, i.e., similar ET value. On the other hand, the ES value of EDMBs is smaller than that of single covalent bonds of comparable polarity. Notice that Manjón and coworkers have shown the electron-deficient nature of the bond in octahedrally-coordinated phases of pnictogens and chalcogens not only using the ES values but also using the charge density and electron localization function (ELF) at the bond critical points between two atoms and, more importantly, by showing that the mechanism of ERMB and EDMB formation is different. A different charge reorganization in the intermediate stage (typical of the formation of multicenter bonds [68]), in which the *trans influence* phenomenon occurs, takes place during the formation of ERMBs and EDMBs [12,13].

In addition to the calculations of ES and ET values in molecules with ERMBs and EDMBs, the supporters of the EDMB model have calculated ES and ET values for solids with ERMBs and EDMBs and have shown that both types of multicenter bonds occur not only in molecules but also in solids with electron-rich elements [12,13]. They have concluded that PCMs, which contain electron-rich elements, feature EDMBs, i.e., electron-deficient bonds, as the supporters of the metavalent bonding model claim. As already commented, this result contrasts with the current belief since EDMBs are only expected to be found in electron-deficient elements [42]. In particular, the supporters of the EDMB model have proposed, in line with Lubchenko and coworkers [64], that linear ERMBs should be defined as 3c-4e bonds because they cannot be extended to linear molecules beyond three centers [12,13]. In other words, multicenter bonds in electron-rich elements with atoms arranged in linear molecules that extend beyond three centers cannot be ERMBs, but EDMBs. The extrapolation of this reasoning to infinite centers leads to the conclusion that extended linear multicenter bonds beyond three centers in solids formed by electron-rich elements must necessarily be EDMBs. The reason for the difficulty of forming ERMBs in linear molecules larger than three centers comes from energetic instabilities and because of the severe violation of the octet rule for the internal atoms of the molecule [12,13,64].

To exemplify these features along one (1D), two (2D), and three (3D) dimensions, the supporters of the EDMB model for PCMs have shown that ERMBs along 1D and 3D can be found in well-known isolated I_3_^−^ and TeI_6_^2−^ molecules within CsI_3_ and Cs_2_TeI_6_ solids, respectively [12]. On the other hand, they have shown that EDMBs can be observed in Sb atoms in infinite linear chains along 2D and 3D in BaZnSb_2_ and the hypothetical sc-Sb, respectively (see Figure 2) [12]. This result contrasts with the previous assumption about the presence of ERMBs in these last two materials that contain an electron-rich elements (Sb) [35,42].

In agreement with the supporters of the hypervalent bonding model in PCMs, Manjón and coworkers have shown that the values of different parameters for the primary and secondary bonds in pnictogens and chalcogens (A7 and A8 structures of pnictogens and chalcogens, respectively) exhibit an inverse relationship as they are compressed until they reach octahedrally-coordinated phases (α-Po and β-Po type phases in pnictogens and chalcogens, respectively) [12,13]. Note that the secondary bonds in the A7 and A8 phases are now categorized as pnicogen and chalcogen bonds by IUPAC. The mentioned inverse relationships evidence the mutual interaction of both primary and secondary bonds, i.e., the *trans influence* caused by the multicenter interaction. This multicenter interaction leads to the formation of the multicenter bonds in the octahedrally-coordinated α-Po- and β-Po-type phases in pnictogens and chalcogens, respectively. Notice that the inverse relationship that evidences the multicenter character of the interaction has been observed not only for the ES and ELF values of both primary and secondary bonds, but also for the bond distances, bond charge densities, and Laplacians of the bond charge density at the bond critical points [12,13]. This result matches the inverse relationship found between the frequencies of optical and acoustic phonons in PCMs when the behavior of a dimerized linear chain under compression is modeled [52].

In summary, the EDMB model for PCMs fully agrees with the metavalent bonding model in the electron-deficient character of the bonds in PCMs and fully agrees with the hypervalent bonding model in the multicenter character of the bonds in PCMs.

Let us comment now on the disagreement of the EDMB model with the hypervalent and metavalent bonding models. The supporters of the EDMB model have shown that the term metavalent is unnecessary to describe bonding in PCMs [12,13]. The reason is that this term is equivalent to the term electron-deficient, as already suggested by Wuttig and coworkers [4,11]. Manjón and coworkers have shown that bonds in PCMs are similar to those present in the bridged B-H-B bonds in B_2_H_6_ and the bonds in sc-As (or sc-Sb) [12,13]. Therefore, the main difference between the metavalent and EDMB models is that the last one clearly claims the multicenter character of the bond. Despite the above considerations, the supporters of the EDMB model consider that the terms metavalent and metavalency are interesting to describe material properties that are intermediate between those of ionocovalent and metallic materials since they present a short and easy way to refer to materials with electron-deficient properties.

**Figure 3:**
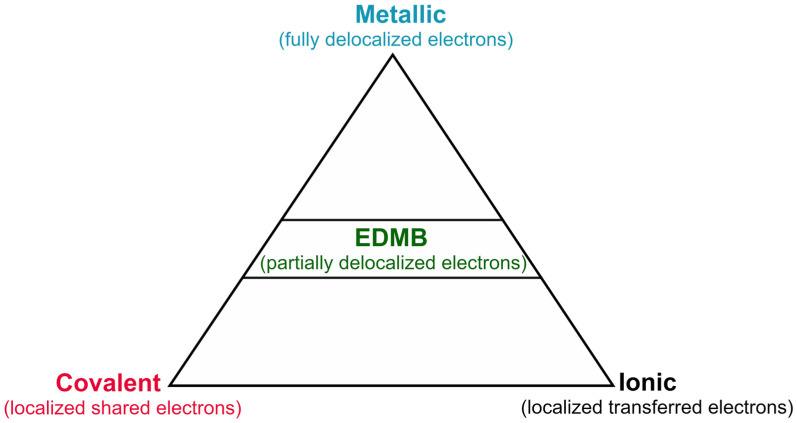
Revisited van Arkel–Ketelaar diagram including covalent, ionic, metallic, and electron-deficient multicenter bondings.

Before going beyond, it must be clarified that the supporters of the EDMB model consider that metallic bonding is indeed characterized by fully delocalized electrons and can be qualified as an electron-deficient bond (less than two electrons localized between two atoms). In other words, metallization is the last step in the process of electron delocalization. However, they consider that metallic bonding cannot be considered a type of multicenter bonding because the metallic bond does not have a directional character, unlike ionocovalent bonding, ERMB, and EDMB. In these last three bonding types, the total or partial electron localization between atoms accounts for the directionality of the interatomic bonding, which in turn is related to the brittleness of covalent materials, the malleability and ductility of metallic materials, and the intermediate character of materials with multicenter bonding, which are well known to exhibit larger and softer bonds than covalent bonds. The above comment on the different types of bonds can be summarized with a revisited van Arkel-Ketelaar diagram (Figure 3), where the EDMB proposed to be present in PCMs is an intermediate bonding between ionocovalent and metallic bondings.

Unlike the supporters of the hypervalent bonding model, the supporters of the EDMB model have proposed that bonds in PCMs should be considered electron-deficient and not electron-rich. Manjón and coworkers have shown that linear bonding motifs with hypercoordinated atoms of electron-rich elements can be found in both ERMBs and EDMBs, e.g., I_3_^−^ units in CsI_3_ and infinite As-As-As bonds (in 3D) in sc-As, respectively [12]. Therefore, they consider that the term hypervalent loses its validity and suggest that this term should be replaced by the term hypercoordinated concerning ERMBs and EDMBs. This suggestion is in line with previous proposals to eliminate the term hypervalent [69,70,71,72,73,74,75,76,77,78,79].

The consideration that electron-rich elements, such as those present in PCMs, could form electron-deficient bonds is now strongly rejected by the supporters of the hypervalent bonding model. For them, the type of bonding is not given by the number of electrons shared between two atoms (around one in PCMs, as shown by the ES values), but by the electron configuration of the involved atoms [42]. In other words, they consider that hydrogen (with a single electron in the valence shell) can form EDMBs, whereas iodine (with seven electrons in the valence shell) cannot. The idea that EDMBs cannot occur in electron-rich elements has been questioned not only by the supporters of the EDMB model [12,13] but also by many other researchers (e.g., see the last chapter in Ref. [40] and already commented refs. [46,47]). In addition, as said above, the position of the supporters of the hypervalent bonding model seems to be at odds with the ERMB nature of bonding in FHF^−^ and the hypercoordination of H in this unit, as already defended by Hoffmann and coworkers [34,42,43,44,45], and with the ES values recently calculated for molecules and solids with ERMBs and EDMBs and plotted in Figure 2 [4,12].

In summary, we believe that the reconciliation of both the metavalent and hypervalent bonding models for PCMs can be reached if: (i) the supporters of the metavalent bonding model assume explicitly the multicenter character of bonding in PCMs, and (ii) the defenders of the hypervalent bonding model assume the electron-deficient character of bonding in PCMs. By considering the above two conditions, the metavalent and hypervalent bonding models lead naturally to the EDMB model proposed in Refs. [12,13].

## 4. ERMBs and EDMBs in Polyiodides and Its Relation to PCMs

This perspective article should end here; however, we consider that the reconciliation of both metavalent and hypervalent bonding models seems to be easier for the supporters of the former than for the defenders of the latter. The reason for this belief is that the supporters of the metavalent bonding model should easily realize the multicenter character of bonding in PCMs because they have reported that GeSe (with *Pnma* structure at room pressure) undergoes a pressure-induced phase transition to the *Cmcm* phase (typical of TlI at room pressure) [62]. Interestingly, while the *Pnma* structure features three covalent bonds (one perpendicular to the layers and two along the layers), the *Cmcm* phase contains both covalent bonds (perpendicular to the layers) and unconventional bonds, like those of PCMs, in the layer plane. Therefore, the supporters of the metavalent bonding model should readily understand that the unconventional bonds formed in the layer plane at the *Cmcm* phase originate from the primary covalent bonds and the secondary noncovalent interactions already present in the layer plane at the *Pnma* phase. The same pressure-induced transformation that occurs in GeSe also occurs in SnSe, where the formation of multicenter bonds from the two mentioned primary and secondary bonds is clearly shown [61]. The mechanism of multicenter bond formation is explained in refs. [12,13]. In this context, Manjón and coworkers have shown that a similar pressure-induced transformation to that of GeSe and SnSe occurs in As_2_S_3_ [53] and in pnictogens and chalcogens [12,13], where EDMBs are formed at high pressure from the original primary and secondary bonds in the low-pressure phase. Therefore, we consider that the assumption of the multicenter character exhibited by the unconventional bond in PCMs is straightforward, as has been previously suggested [22,24,52].

On the contrary, we believe it is going to be harder for the supporters of the hypervalent bonding model to agree with the electron-deficient character of the bonding in PCMs. The reason is that the formation of electron-deficient bonds in molecules and solids composed of electron-rich elements seems not to have been sufficiently justified in the literature yet, except for molecules in a few of the above-commented works [40,46,47] and solids only in Refs. [12,13].

Since we consider that the supporters of the hypervalent bonding model for PCMs might have notable difficulties in changing their minds, we want to provide, in this last section, a few examples to illustrate how electron-rich elements can form both linear ERMBs and linear EDMBs. In particular, we will show how iodine atoms can form linear ERMBs in three-center molecules (I_3_^−^) and linear EDMBs when the molecule extends beyond three centers, as is the case of infinite linear iodine chains (I_∞_). For this purpose, we will make use of theoretical calculations already discussed in the literature. Moreover, we will make the connection between the bonding in PCMs, such as cubic GeTe, and the bonding in polyiodides, following the idea developed in Ref. [14]. In this way, we will show that (i) the different types of bonding in polyiodides depend on the size and geometry of the polyiodides, and (ii) cubic GeTe and I_∞_ are exclusively characterized by EDMBs, unlike other polyiodides. We hope these examples will help the supporters of the hypervalent bonding model consider their position regarding the electron-deficient character of bonding in PCMs.

**Chart 1:**
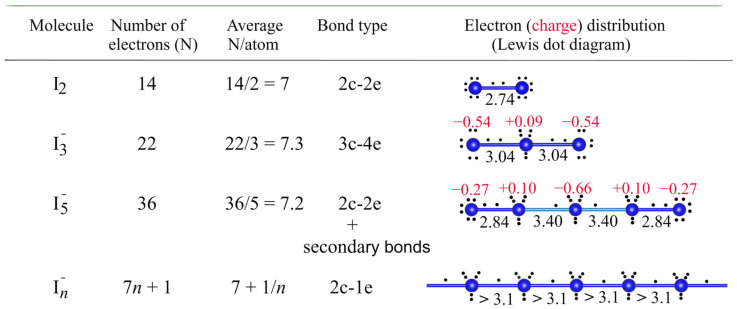
Bonds in linear I_2_, I_3_^−^, I_5_^−^, and I_n_^−^ molecules. (**Left**) Electron counting in linear iodide molecules according to Hoffmann’s rules [35]. (**Right**) Bond types and electron distribution in atoms (blue circles) and bonds (blue lines) according to the Lewis dot model (black dots represent electrons) considering the ab initio restricted Hartree-Fock simulations of Sæthre et al. for I_2_, I_3_^−^, and I_5_^−^ with the corresponding atomic charges (in red, in e) and bond distances (in black, in Å) [80].

To discuss the different natures of covalent bonds, ERMBs, and EDMBs in polyiodides, we show in Chart 1 several linear polyiodides that will help us to understand the formation of ERMBs in a linear three-center molecule, such as I_3_^−^, and of EDMBs in the infinite linear I_n_^−^ molecule, when n tends to ∞. Notice that Chart 1 makes use of the electron counting reported by Papoian and Hoffmann to justify the formation of Sb−Sb−Sb ERMBs in the infinite linear Sb^2−^ chains of Li_2_Sb [35]. We will show that, despite the electron counting conducted by Papoian and Hoffmann being correct, the explanation of the bonding in the infinite linear Sb^2−^ chain was not correct. For the sake of our convenience, we plot in Chart 1 linear molecules of iodine atoms (isoelectronic to Sb^2−^ atoms) because they can be nicely interpreted by Lewis dot diagrams with the help of already published restricted Hartree-Fock (RHF) calculations for the linear molecules of several I_n_^−^ atoms [80]. The Gaussian basis set used in the RHF calculations is similar to the current GTO 6-311G* basis set [81] and has been properly tested for iodine [82]. We will show that the description provided by RHF calculations has been confirmed by more modern calculations.

RHF calculations [80] correctly predict that the I_2_ molecule, with a total charge of 14 electrons (average of 7 electrons per atom), has a bond distance of 2.74 Å, which is similar to the experimental bond distance [83]. Let us now distribute the number of electrons in the molecule according to the Lewis dot diagram. For that purpose, we first place two electrons in each lone electron pair (LEP) and then distribute the remaining electrons among the available bonds. Only by placing electrons in this way, which considers first the explanation of the geometry and then of the bonding, which could be odd to many chemists, can we find covalent bonds, ERMBs, and EDMBs while correctly explaining the geometries of angular and linear bonds in materials, as already pointed out [46,47]. Thinking in this way, six of the seven electrons of each I atom are first distributed in three LEPs around each I atom, and the seventh electron is shared between the two I atoms to form a single covalent 2c-2e bond in I_2_.

Let us now consider the linear I_3_^−^ anion with a total charge of 22 electrons (average of 7.3 electrons per atom). RHF calculations for the linear I_3_^−^ anion yield iodine atoms separated by an equal bond distance of 3.04 Å, i.e., ca. 0.3 Å larger than the 2c-2e bond in I_2_ [80]. This result is again in good agreement with the average value of experimental data found in the Cambridge Structural Database for this anion (ca. 2.92 Å) [83]. The larger value of the I-I bond distance in I_3_^−^ than in I_2_ and the different charge distribution between the central and external atoms in I_3_^−^ obtained in RHF calculations were confirmed by more modern calculations [84,85,86,87]. Based on the above considerations, we can distribute electrons according to the above-proposed procedure by first placing six of the seven electrons of each I atom into three LEPs around iodine atoms for a total of 18 electrons. The remaining four electrons of the I_3_^−^ molecule must then be shared by two pairs of iodine atoms in the two colinear bonds. As expected for a 3c-4e ERMB, theoretical calculations suggest that the negative charge of the I_3_^−^ anion is concentrated on the external atoms and the ERMBs are larger than the covalent bonds. Therefore, in Chart 1, we have placed one electron in the middle of each bond (shared by two atoms) and another electron much closer to the external atoms. In this way, this electron distribution nicely explains why the external atoms have a larger charge than the central one. In addition, this electron (closer to the external atom) is only partially shared by the two neighboring atoms and explains why the ES values between two atoms in 3c-4e bonds have been found typically smaller than those of non-polar 2c-2e bonds, larger than those of polar 2c-2e bonds with the same ET (polarity), and much larger than those for 3c-2e EDMBs, such as the central B-H-B bonds of B_2_H_6_ [4,12]. The much larger ES value of a 3c-4e bond, like I_3_^−^, than the ES value of a 3c-2e bond, like B_2_H_6_, justifies once again that ERMBs are not 2c-1e bonds, unlike the 3c-2e bonds. Notice that the smaller ES value of the 3c-4e bond than of the 2c-2e bond agrees with the smaller bond order and the smaller value of the stretching vibrational frequencies in I_3_^−^ than in I_2_ [88].

Let us now explain the linear I_5_^−^ anion with a total of 36 electrons (an average of 7.2 electrons per atom). RHF calculations show that there are two very different bond distances: 2.84 Å (slightly larger than that of the covalent bond in I_2_) and 3.40 Å (much larger than that of the 3c-4e bond in the I_3_^−^ anion) [80]. The shorter value of the external bonds than the internal bonds in I_5_^−^ anions (either in linear or bent geometry) was confirmed by modern calculations [85,86,88,89] that are in relatively good agreement with experiments [83]. One could think at first glance that an electron-rich 5c-6e bond could be formed in the linear I_5_^−^ anion, as would be expected by the supporters of the hypervalent bonding model in PCMs. Such a hypothesis would lead to a scheme of molecular levels similar to those plotted for the 3c-4e bonds in Figure 1 (right) but with five levels instead of three. In that system, the first three levels (two bonding and one nonbonding) would be filled with electrons, and the two last levels (antibonding) would be empty. However, the bond distances and the electron distribution found in the simulations do not support the formation of a 5c-6e ERMB [80,88,89]. According to RHF simulations summarized in Chart 1, we should distribute the electronic charge at the I_5_^−^ anion in an unequal way along the atoms in the chain, with the largest charge located at the central atom.

We propose the following electron distribution for the simulated linear I_5_^−^ anion (see Chart 1): The first six electrons are distributed into three LEPs around each iodine atom, yielding 30 electrons, and the remaining six electrons of the molecule are distributed between the four bonds of the linear I_5_^−^ molecule. Since the central atom bears the largest electronic charge and the longest bonding distances, and the external bonds are almost as short as covalent bonds, the external bonds should share two electrons, whereas the long bonds involving the central atom should share only one electron. In addition, the large charge and bonds of the central atom clearly suggest that the electrons of the surrounding bonds should be close to the central atom. Therefore, according to the RHF simulations and the electron distribution we have provided, the linear I_5_^−^ anion in Chart 1 can be interpreted as formed by two external I_2_ molecules weakly linked to a central I^−^ anion through secondary bonds in a [(I^−^)·2I_2_] configuration. Noteworthy, this configuration agrees with the interpretation of this polyiodide [83,88,89,90]. The slight enlargement of the external covalent bonds in the linear I_5_^−^ anion is caused by the *trans influence* of the weak secondary bonds (now considered to be halogen bonds according to IUPAC) on the primary covalent bonds. Moreover, the proposed electron distribution (with fewer electrons in the internal bonds than in the external ones) is in good agreement with the simulated vibrational modes of the I_5_^−^ anion since the symmetric stretching modes of the external I-I bonds have much larger frequencies (165.0 cm^−1^) than those of the internal I-I bonds (55.5 cm^−1^) [89].

In summary, the previous reasoning shows that ERMBs do not exist in the linear I_5_^−^ anion, unlike in the linear I_3_^−^ anion. This interpretation is also in line with the studies of Sæthre et al. [80], who commented on the weakness of the central bonds in the linear I_5_^−^ anion and the preference of this anion for the bent V or L geometries instead of the linear one, as it has indeed been experimentally observed [33,83,88,91]. A more detailed explanation of the different geometries preferred by the most known polyiodides will be published elsewhere.

At this point, we want to remark that we have preferred the use of the term secondary bond in the previous paragraph as it possesses a broader group-wise meaning than the term halogen bond since ERMBs and EDMBs (based on the equalization of primary and secondary bonds) occur in many elements across the periodic table [12,13]. We consider that neither the halogen bond nor the secondary bond views are fully adequate to describe the bonding in the linear I_5_^−^ anion. On the one hand, the halogen bond is linked to a nucleophile/electrophile interaction, according to IUPAC recommendation [92], forcing one to formally tear apart a well-defined molecule and picture it as arbitrary nucleophilic or electrophilic non-factual fragments (an always possible yet needless exercise). On the other hand, weak bonds in I_5_^−^ involve valence electrons, so they cannot be considered mere “secondary Lewis acid-base interactions”, as in the original secondary bonding proposition [93]. These comments are in line with the current criticism of group-by-group electrophile nomenclature (halogen-, chalcogen-, pnictogen-, etc.…- bonds) and demands for a proper systematization of supramolecular interactions [94,95]. While surely interesting, the discussion of these topics is beyond the scope of the present contribution.

Now we arrive at the final and most important point in Chart 1, the linear I_n_^−^ molecule. The bonding type in this linear molecule, when n tends to ∞, is similar to that present in PCMs, such as cubic GeTe, since both feature an infinite linear chain of bonds. The linear I_n_^−^ molecule has a total of 7n + 1 electrons (average of 7 + 1/n electrons per atom). Thus, an infinite molecule should have ca. 7 electrons per atom, as in the I_2_ molecule. This result agrees with the electron counting of Papoian and Hoffmann (see Section 4.2. in Ref. [35]). In other words, it does not matter if an infinite linear molecule is I_∞_^−^, I_∞_, or I_∞_^+^ since, in these cases, the extra charge can be neglected in all instances. To our knowledge, ab initio calculations for the isolated I_∞_ molecule have not been published yet; however, infinite linear halogen chains have been theoretically predicted by ab initio simulations [96,97] and experimentally found in the recently reported cubic *Pm*-3*n* phase of *AX*_3_ compounds (A = Na, K, Rb, Cs; X = Cl, Br, I) [96,98,99]. In addition, it must be stressed that infinite quasi-linear iodine chains have been recently reported in several compounds at room temperature, such as the Pyrroloperylene–Iodine Complex [100] and the phenanthrolinium salt [101]. In this context, we want to stress that infinite linear atomic chains have also been found at room pressure in some solids with pseudo-halogen atoms, e.g., Te^−^ atoms in the *4a* Wyckoff site in TlTe [35,102] and Sb^2−^ atoms in the *1b* Wyckoff site in Li_2_Sb [35,103], since both Te^−^ and Sb^2−^ atoms are isoelectronic to I atoms. Notice that all the above-mentioned Te (Sb) atoms should have the same electronic charge as TlTe (Li_2_Sb) because they are all equivalent (occupying the same Wyckoff position).

Importantly, ab initio simulations on *AX*_3_ compounds have emphasized the different bonding and properties of the phases with *X*_3_^−^ molecules (*Pnma* and *P*-3*c*1) and the phase with linear *X*_∞_ chains (*Pm*-3*n*) [96,98,99]. The different properties of those phases are clear if we consider that *X* atoms are bonded by ERMBs in phases with *X*_3_^−^ molecules, whereas *X* atoms are bonded by EDMBs phases with linear *X*_∞_ chains. In particular, it has been shown that the phases with isolated *X*_3_^−^ molecules have insulating properties while the phases with linear *X*_∞_ chains have metallic properties due to the presence of delocalized electrons [99], as would be expected for a half-filled band as the infinite linear H chain or sc-As [12,13,104,105]. The recent results on *AX*_3_ compounds with phases containing *X*_3_^−^ molecules fully agree with calculations of ERMBs in I_3_^−^ molecules that have a considerable bandgap, making these units insulating [106]. In summary, the already performed ab initio calculations give support to the hypothesis that the bonding in the linear *X*_∞_ chain with delocalized electrons, as in PCMs and sc-As [12,13], has a different nature and properties than the ERMB in the *X*_3_^–^ molecules.

The aforementioned features lead us to propose the following electron distribution for the infinite linear I_n_^−^ chain shown at the bottom of Chart 1. As in the previous cases, six of the seven electrons would be distributed in three LEPs around each I atom. According to the VSEPR theory [107], the LEPs will be located in the plane perpendicular to the linear chain (as in the central atoms of the linear I_3_^−^ and I_5_^−^ chains), and the seventh electron will be shared between two contiguous atoms. Therefore, there will be a 2c-1e bond between every two I atoms in the infinite linear chain (see bottom of Chart 1), with all atoms having the same charge and being equally separated. This means that extended linear molecules, irrespective of their charge, feature a 2c-1e bond, i.e., the extended analog of the 3c-2e EDMBs of bridging B-H-B atoms in B_2_H_6_ [11,12,40].

The 2c-1e bonds have much smaller ES values than for ERMBs and covalent bonds, so a larger bond length for EDMBs than for ERMBs and covalent bonds is expected [12,13]. This hypothesis is supported by the bond lengths observed in the Pyrroloperylene–Iodine Complex [100] and the phenanthrolinium salt [101]. In both compounds, an average I-I bond distance of 3.15 to 3.25 Å has been found. Therefore, we propose a bond length higher than 3.1 Å for 2c-1e bonds in polyiodides (see bottom of Chart 1). A similar trend to that of polyiodides was observed for Te^−^ atoms in the *4a* Wyckoff site in TlTe [35,102] and for Sb^2−^ atoms in the *1b* Wyckoff site in Li_2_Sb [35,103]. The Te(*4a*)–Te(*4a*) and the Sb(*1b*)–Sb(*1b*) bonds in the infinite linear atomic chains have longer bonds (above 3.0 Å) than their equivalent covalent bonds in elemental Te and Sb (below 3.0 Å). These results are in line with the estimation of Pauling, who considered that one-electron bonds (electron-deficient bonds) should be ca. 0.3 Å longer than single covalent 2c-2e bonds [108].

In summary, we have schematically shown for 1D that the infinite linear atomic chains in solids, such as those present in TlTe, Li_2_Sb [12], and several halides, should have a 2c-1e bond that is the extended analog of a 3c-2e EDMB despite being formed by an electron-rich element, such as iodine or pseudo-iodine atoms (Te^−^ and Sb^2−^). These 2c-1e EDMBs have similar features to those of the infinite linear chains of Te-Ge-Te-Ge… along the three spatial directions in cubic GeTe [1]. We also notice that a homogeneous distribution of distances and charges, as the one we propose here for the linear I_∞_ chain, has been theoretically found in linear chains of Br atoms confined in a zeolite when compressed above 23 GPa [109] and suggested in I atoms in the same environment [110].

In this context, we want to stress that the supporters of the EDMB model have suggested that ERMBs cannot be found in molecules longer than three centers because it results in an energetically unstable system and would lead to a severe violation of the octet rule [12,13]. This result is in line with the suggestions of Lubchenko and coworkers [64]. Indeed, examples of linear molecules longer than three centers and having ERMBs have not been found yet, at least among isolated polyiodides. Note that linear polyiodides longer than three centers typically present a bent geometry instead of a linear one, as already commented for the linear I_5_^−^ molecule [80,83]. Indeed, bending polyiodides longer than three centers seems to be necessary to hold ERMBs. A full discussion regarding the different geometries in the most common polyiodides and their different covalent bonds, ERMBs, and EDMBs will be published elsewhere.

It is interesting to notice that the instability of linear polyiodides larger than I_3_^−^ is supported by recent results. It is known that linear polyiodides longer than three centers have been found in several compounds, like zeolites, metalorganic frameworks, and carbon nanotubes, which have linear cavities that might act as templates and electron reservoirs for the formation of linear molecules [106,111]. Recent works suggest that linear I_3_^−^ molecules can be formed inside linear cavities when electrons are supplied to the iodine chains [106] and that linear I_5_^−^ molecules inside single-wall carbon nanotubes transform into I_3_^−^ molecules when electrons are added to the system (the reaction can be thought to be 3I_5_^−^ + 2e^−^ → 5I_3_^−^) [111]. Noteworthy, a similar behavior of transformation of I_5_^−^ anions into I_3_^−^ anions has been observed under pressure [112]. All these results not only support the instability of polyiodides longer than three centers but also support the equivalence between pressure and reduction (adding electrons to a system) already commented on Ref. [12] concerning the formation of multicenter bonds. This equivalence can be understood if we consider that both pressure and reduction result in an increase in electron density that promotes the formation of multicenter bonds [12].

**Figure 4:**
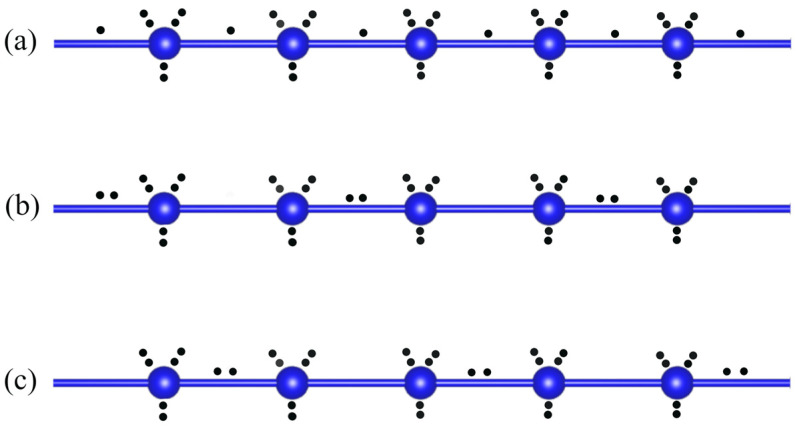
Distribution of electrons (black dots) in the atoms (blue circles) and bonds (blue lines) of (**a**) the infinite linear chain of I atoms (I_∞_) according to the Lewis dot model. The infinite linear iodine chain can be considered to result from the resonance of chains (**b**,**c**) with alternate 2c-2e bonds and no bonds.

At this point, it is interesting to note that Lipscomb commented that the 3c-2e bond of boranes could be considered the sum of two linked or interacting 2c-1e bonds [113]. It must be noted that, alternatively, the 2c-1e bonds of the linear I_∞_ chain can be considered, within the valence bond theory, as a resonant 2c-2e bond oscillating between two adjacent ionocovalent bonds, such as those of Figure 4. This consideration also reinforces the multicenter character of this kind of bond. As already commented, we expect the I-I bond distance in the linear I_∞_ chain to be larger than 3.1 Å, due to the smaller ES value of EDMBs than ERMBs and covalent bonds.

In the last part of this work, we want to highlight the different bonding between infinite linear iodine chains and infinite zigzag iodine chains to show how bending can make a difference in the bonding of polyiodides. In a recent paper, the bond in a PCM, cubic GeTe, has been compared to the bond in polyiodides [14]. We have already commented that GeTe shows the same type of bonding as the linear I_∞_ chain. Now we will see that it has a completely different bond than that of the infinite zigzag iodine chain. This result can be understood because the different polyiodides have different kinds of bonds depending on the polyiodide size and geometry [83].

**Chart 2:**
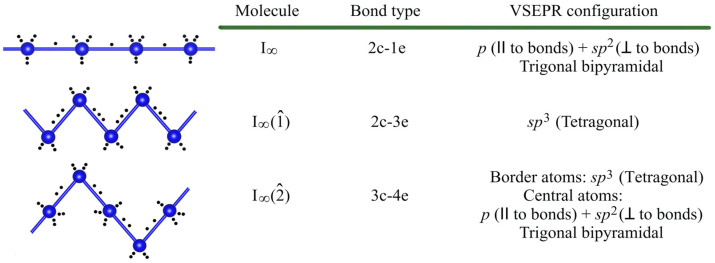
Distribution of electrons (black dots) in the atoms (blue circles) and bonds (blue lines) in the infinite linear iodine chain, I_∞_, and the infinite zigzag (or angular) iodine chains, I_∞_($\hat{1}$) and I_∞_($\hat{2}$), according to the Lewis dot model (**left**). The bonding type, hybridization, and geometry of these chains according to VSEPR rules are also indicated (**right**).

In Chart 2, we just show three types of infinite iodine molecules: the infinite linear iodine chain (I_∞_), the infinite zigzag (or angular) iodine chain bent at every atom, I_∞_($\hat{1}$), and the infinite zigzag iodine chain bent every two atoms, I_∞_($\hat{2}$). As previously commented, I_∞_ is characterized by each iodine atom having six electrons, forming three pairs of nonbonding LEPs that are perpendicular to the bonds along the linear chain. The remaining electron of the iodine atom is shared between the two next-neighbor atoms of the linear chain in a p-type geometrical configuration. In this way, each iodine in I_∞_ shows a total trigonal bipyramidal configuration with three LEPs being distributed in a sp^2^ geometrical configuration that minimizes the electronic repulsion between the three LEPs and the bonding electrons along the linear chain according to the VSEPR theory [72,107]. It must be noted that a similar configuration was proposed for the methanonium or methanium CH_5_^+^ ion with electron-deficient bonds (Figure 7.1 in Ref. [40]). In summary, there is only one electron shared (EDMB) between two iodine atoms in I_∞_, as already explained in Chart 1.

In the I_∞_($\hat{1}$) chain, the angular geometry forces us to consider that each I atom has four electrons distributed in two terminal LEPs pointing outwards of the chain, and the three remaining electrons should be shared between the two next-neighbor iodine atoms. In this infinite zigzag chain, all I atoms show a sp^3^ VSEPR configuration to minimize the electronic repulsion. It can be noted that this configuration (with three electrons per bond) would result in an important violation of (i) Pauli’s exclusion principle (there are three electrons shared between two atoms, i.e., in the same space region) and (ii) the octet rule for all atoms. This reasoning means that it is highly improbable that this chain could be observed in halogens unless there is an important charge transfer (one electron per atom) from this unit to another one, as it seems to be the case of infinite linear iodine chains inside carbon nanotubes [106]. Instead, this configuration can be observed in chalcogens [114], since they have one valence electron less than halogens. This type of infinite zigzag atomic chain has been observed in the Zintl phases BaSn [115] and EuSb_2_ [35] because, according to the Zintl–Klemm concept, both Sn^2−^ and Sb^−^ are Ψ-Te (pseudo-Te) atoms and show the same bonding behavior as Te; that is, they form two covalent 2c-2e bonds to satisfy the 8-N rule. Note that the infinite zigzag Sb chains in EuSb_2_ (also Sn in BaSn) with two electrons shared between two atoms are typical covalent bonds, as suggested by their Sb–Sb bond lengths (ca. 2.93 Å) in comparison with the Sb–Sb bond lengths (ca. 3.24–3.26 Å) of EDMBs in BaZnSb_2_ [12,116] and in Li_2_Sb [103].

On the other hand, I_∞_($\hat{2}$) exhibits two types of I atoms. Central atoms have the same configuration as those of I_∞_, while external atoms have the same configuration as those of I_∞_($\hat{1}$). As observed in Chart 2, I_∞_($\hat{2}$) is characterized by sharing two electrons between every pair of atoms, thus resulting in 3c-4e bonds at each linear fragment of the infinite zigzag chain. In other words, I_∞_($\hat{2}$) is a condensation of three-center molecules each with a 3c-4e ERMB and hypothetically could be formed by elemental iodine at high pressure, as recently simulated and discussed [14]. This type of infinite zigzag chain has also been observed in the Zintl phases of tellurides, such as UTe_5_ [117].

To finish this discussion, we want to highlight that it is curious that ERMBs in short molecules, i.e., forming 3c-4e bonds (e.g., in I_3_^−^), exhibit a linear or quasi-linear geometry, while in large molecules (e.g., the infinite zigzag chains of Te^–^ in UTe_5_ and of I in the hypothetical I_∞_($\hat{2}$) chain), form bent geometries. On the contrary, EDMBs in short molecules, i.e., forming 3c-2e bonds (e.g., in H_3_^+^ or B_2_H_6_), are mainly characterized by bent geometries, while in large molecules (e.g., I_∞_), they have a linear or quasilinear geometry. This observation points to the existence of a kind of anti-symmetry between ERMBs and EDMBs.

All in all, we have provided here a new physical insight by which the infinite linear iodine chain (and the corresponding chains of their equivalent isoelectronic atoms) features a 2c-1e EDMB that can be considered the extended version of the 3c-2e bond. Moreover, we have shown with a simple pen-and-paper exercise that not only is the I_∞_($\hat{1}$) impossible to find in isolated halides but also that the bonding of the linear I_∞_ chain (with EDMBs) is different from that of the zigzag I_∞_($\hat{2}$) chain (with ERMBs). Therefore, we conclude that all bonds in polyiodides are not equal; their nature depends on the size and geometry of the chain. Consequently, we can conclude that the bonding in rocksalt-type PCMs, such as cubic GeTe, is comparable to that of the I_∞_ chain but completely different to that of the I_∞_($\hat{2}$) chain.

## 5. Conclusions

This paper intends to provide an actual perspective of the chemical bonding in PCMs by commenting on the three current chemical bonding models, which try to explain their unconventional bonds, their structures (including atomic hypercoordination), and their extraordinary properties: the hypervalent (electron-rich multicenter), the metavalent (electron-deficient), and the electron-deficient multicenter bonding models. In particular, the agreements and disagreements between the three different bonding models have been commented on. Moreover, we have provided a new physical insight by which the third model (the electron-deficient multicenter model) can result in the reconciliation of two previous opposite models (the hypervalent and metavalent models). The reason is that the third model considers the pros and cons of both previous models.

In brief, the success of the metavalent bonding model is that bonds in PCMs are soft and directional electron-deficient bonds, while its pitfall is that it does not explicitly consider the multicenter character of the 2c-1e bond. When the multicenter character of the bond is considered, the metavalent bonding model becomes naturally the electron-deficient multicenter bonding model. Therefore, the term metavalent is no longer necessary to define the bonding since the bonding in PCMs is not a brand-new bonding type. On the other hand, the success of the hypervalent (electron-rich multicenter) bonding model resides in the demonstration of the multicenter character of the bonds in PCMs that leads to hypercoordinated linear bonding motifs in PCMs. Since these linear motifs can be found in molecules and solids with both electron-rich and electron-deficient elements [12,13], the term hypervalent is no longer valid and should be replaced by hypercoordinated. In other words, the main pitfall of the hypervalent bonding model is that it considers that the bond in PCMs is electron-rich and not electron-deficient. If this consideration is reversed (note that the formation mechanisms of ERMBs and EDMBs are different [12,13]), the electron-rich multicenter bonding model also becomes, in a natural way, the electron-deficient multicenter bonding model.

To help the defenders of the hypervalent bonding model reconsider their position, we have reasoned about the different types of bonding in different polyiodides depending on their size (number of atoms involved) and geometry (linear or bent), which have recently been commented on concerning the bonding in cubic GeTe [14]. In particular, it has been recently suggested that linear molecules longer than three centers cannot hold ERMBs (bending is needed), as already discussed [12,13,64]. Moreover, it has also been reasoned that the infinite linear iodine chain should feature an EDMB, like cubic GeTe, and unlike infinite zigzag iodine chains.

At this point, we want to comment that the current state of ideas concerning the bonding in PCMs reminds us of the words attributed to W.L. Bragg: “The important thing in science is not so much to obtain new facts as to discover new ways of thinking about them” [118]. In conclusion, we have shown here that a reconciliation of both the metavalent and hypervalent (electron-rich multicenter) bonding models for PCMs is possible if we think differently and conclude that PCMs are governed by the old, known electron-deficient multicenter bonding.

We want to finish by stressing that the electron-deficient multicenter bonding model proposed in Refs. [12,13] not only allows one to explain the structure and properties of PCMs, such as minerals galena, clausthalite, altaite, and tetradymite, but also the structure and properties of some minerals at high pressure, such as orpiment (As_2_S_3_), stibnite (Sb_2_S_3_), and bismuthinite (Bi_2_S_3_), which show hypercoordinated atoms at high pressure. Moreover, the electron-deficient multicenter bonding model allows for the explanation of the structure of many materials, including complex Zintl phases, intermetallics, and cluster compounds [12]. The information obtained for multicenter bonds in high pressure studies, such as Refs. [12,13], proves that high pressure is an extraordinary tool that promotes hypercoordination and allows to study the two different kinds of multicenter bonds, either ERMBs or EDMBs [12,13,53,54,55,56,57,58,59,60,61,62,63,64,96,97,98,99].

## Data Availability

No new data were created or analyzed in this study.
